# Predictive value of NLMR, PLR, and ferritin in relation to SOFA, APACHE II, and SAPS II in sepsis patients

**DOI:** 10.62838/jccm-2026-0009

**Published:** 2026-04-30

**Authors:** Oana Coman, Bianca-Liana Grigorescu, Adina Hutanu, Stefania Raluca Fodor, Marius Petrisor, Leonard Azamfirei

**Affiliations:** Department of Simulation Applied in Medicine, George Emil Palade University of Medicine, Pharmacy, Science, and Technology of Targu Mures,Romania; Department of Anaesthesiology and Intensive Therapy, George Emil Palade University of Medicine, Pharmacy, Science, and Technology of Targu Mures,Romania; Department of Laboratory Medicine, George Emil Palade University of Medicine, Pharmacy, Science, and Technology of Targu Mures,Romania; Centre for Advanced Medical and Pharmaceutical Research, Immunology, George Emil Palade University of Medicine, Pharmacy, Science, and Technology of Targu Mures,Romania

**Keywords:** sepsis, septic shock, NLMR, PLR, severity scores

## Abstract

**Introduction:**

Sepsis, a critical topic in the medical field, remains one of the deadliest pathologies in intensive care units. It involves an overzealous immune system, with a hyperinflammatory phase that overlaps with a subsequent hypoinflammatory phase.

**Aim of the study:**

To ease the burden on medical systems, this study aimed to assess the predictive value of clinical severity scores (Sequential Organ Failure Assessment (SOFA), Acute Physiology and Chronic Health Evaluation II (APACHE II) score, and the Simplified Acute Physiology Score II (SAPS II)) and inflammatory biomarkers (neutrophile-to-lymphocyte-to-monocyte ratio (NLMR), and platelet-to-lymphocyte ratio (PLR), carboxyhemoglobin (COHb) and ferritin) in predicting outcomes of critically ill intensive care unit (ICU) patients.

**Material and methods:**

This prospective, observational study included 101critically ill patients, for whom we assessed the parameters on the first and fifth days after confirmation of either sepsis or septic shock in ICU, according to the Sepsis-3 Consensus.

**Results:**

Severity scores showed significant correlations on both day 1 and day 5 across all groups. APACHE II and SAPS II correlated with ferritin on day 5 in sepsis, septic shock, and non-survivors. The severity scores correlated with COHb on day 5 in survivors, and on day 1 in non-survivors. NLMR and PLR correlated consistently across groups, with additional associations between these ratios, ferritin, and COHb, particularly in non-survivors. Regarding mortality, NLMR on day 1 showed only modest predictive value, which declined to non-significant by day 5. In contrast, the SOFA, APACHE II, and SAPS II scores demonstrated good discriminatory ability on both days, confirming their strong and reliable performance in predicting mortality.

**Conclusions:**

The study shows that simple cellular ratios and severity scores correlate with ferritin, COHb, and each other, reflecting inflammation, oxidative stress, and organ dysfunction in sepsis. Because these markers are inexpensive and easy to monitor, they may enhance bedside risk stratification, though broader prospective studies are still required.

## Introduction

Sepsis is a critical topic in the medical field and remains one of the deadliest pathologies in intensive care units (ICU) around the world. It involves an overzealous immune system and a complex interplay between a hyperinflammatory phase, overlapped at some point by a hypoinflammatory phase. Sepsis is characterized by a syndrome of physiological, pathological, and biochemical abnormalities caused by an infection, prompting circulatory dysfunctions, cellular metabolism disruption, organ function damage and failure, and death [1]. It refers to life-threatening organ dysfunction resulting from a dysregulated host response to that infection [2].

In 2020, a research by Rudd KE et al. studying sepsis incidence and mortality revealed a 18.8% decrease in sepsis cases between 1990 and 2017, with reported deaths dropping from 15.7 million in 1990 to 11 million in 2017 [3]. Despite this reduction, sepsis continues to pose a significant burden on the medical community. As a response, the World Health Organization (WHO) published its first global report on the epidemiology and impact of sepsis in 2020. This report emphasizes the need for physicians and other healthcare professionals to develop tools for the rapid diagnosis and monitoring of sepsis progression [4].

There is considerable variability in sepsis-related costs between different countries. A systematic review from 2022 found that the costs associated with sepsis per patient range from €1.101 to €91.951 [5]. The aging population, preexisting comorbidities, and acute complications, along with the costs associated with investigations, treatment, hospitalization, and long-term rehabilitation of patients, make sepsis a condition that is not only clinically life-threatening but also an economic burden with a significant impact on healthcare worldwide.

The implementation of the Sepsis Bundle has led to substantial advancements in the treatment of both sepsis and septic shock, aiming to reduce mortality rates [1]. However, proper monitoring is challenging. Therefore, there is a need to develop faster, practical, and reliable assessment tools that enable early identification of critically ill patients with sepsis and improve treatment success rates. However, the profound interindividual variability in immune dysfunction among patients with sepsis requires stratification using inflammatory biomarkers, which provide critical insights into the underlying immune dysregulation. This approach, referred to as predictive enrichment, is essential for tailoring immunotherapeutic interventions to individual patient profiles and optimizing clinical outcomes [6, 7].

The Sequential Organ Failure Assessment (SOFA) score, recommended by the Sepsis-3 Consensus, is commonly used to assess the severity of organ dysfunction [2]. However, other severity scores have been used in conjunction with the SOFA score to gain a better understanding of disease progression. In a previous study, we found a significant correlation between SOFA score, the Acute Physiology and Chronic Health Evaluation II (APACHE II) score, and the programmed death axis, offering a complex image of the intricate changes occurring during sepsis [8].

Therefore, identifying more straightforward and more practical approaches for prognostic assessment in patients with septic shock remains essential. The complete blood count (CBC) offers relevant information regarding the immunological status of sepsis, enabling the calculation of various ratios that reflect both innate and adaptive immune responses. The neutrophil-to-lymphocyte ratio (NLR), the lymphocyte-to-monocyte ratio (LMR), and the platelet-to-lymphocyte ratio (PLR) have been used as prognostic biomarkers in sepsis, proving their reliability in indicating systemic inflammation, physiological stress, as well as the interplay between thrombotic and inflammatory responses [9,10,11,12,13].

Hepatic involvement represents an important component of sepsis progression, with impaired hepatocyte-mediated bilirubin clearance and elevated transaminase levels reflecting the severity of liver dysfunction [14]. In this context, enhanced heme degradation via the heme oxygenase-1 (HO-1) pathway leads to increased hepatic production of carbon monoxide, detected in blood as carboxyhemoglobin (COHb) and exhaled CO [15].

Alongside procalcitonin and C-reactive protein, ferritin and COHb can also be used as inflammatory markers to track the progression and prognosis of sepsis. Elevated ferritin levels arise from excessive inflammation and contribute to the inflammatory process by binding with T-cell immunoglobulins and promoting the expression of various pro-inflammatory mediators [16,17,18,19].

We set out to identify a panel of prognostic indicators based on simple, cost-effective, readily available laboratory markers. Therefore, this study aims to assess the predictive value of clinical severity scores (SOFA, APACHE II, and the Simplified Acute Physiology Score II (SAPS II)) and inflammatory biomarkers (neutrophile-to-lymphocyte-to-monocyte ratio (NLMR), and PLR, COHb and ferritin) in determining the clinical outcome of critically ill patients admitted to the ICU. Our goal is to identify and screen high-risk patients within an appropriate timeframe.

## Materials and methods

### Ethical Approval

We conducted a prospective observational single-centre study including 101 adult patients, diagnosed with sepsis, who required intensive care unit admission at the County Emergency Clinical Hospital in Târgu Mures, Romania, over the period from 7^th^ July 2021 to 12^th^ March 2023. The protocol complied with the ethical standards outlined in the Declaration of Helsinki of 1975. Ethical approval was granted by the Ethics Committee of the University of Medicine and Pharmacy, Science, and Technology “George Emil Palade” of Târgu Mures, (Mures, Romania; approval no 1425/01.07.2021). Written informed consent for participation and publication of anonymized data was obtained from each patient or their legally authorised representative, and all procedures were conducted in accordance with the General Data Protection Regulation.

### Study Cohort

#### Inclusion criteria

The study population consisted of patients aged 18 and above who met the Sepsis-3 Consensus criteria for sepsis or septic shock [2]. We divided the patients into four subcategories according to diagnostic (sepsis and septic shock) and survival status (survivors and non-survivors).

#### Exclusion criteria

Exclusion criteria comprised active oncological disease under chemotherapy or radiation therapy, chronic liver dysfunction, use of systemic corticosteroids or immunosuppressive medication, and the presence of autoimmune conditions.

### Evaluated Parameters

Clinical data were systematically recorded for all enrolled patients and encompassed three main domains: (1) demographic and baseline characteristics, including age, sex, body mass index, infection source, and pre-existing comorbidities; (2) the laboratory parameters obtained within the first 24 hours following ICU admission, comprising complete blood count components (neutrophils, lymphocytes, monocytes, platelets, and haemoglobin), arterial blood gas measurements, ferritin levels, and additional biochemical markers such as serum lactate, total bilirubin, creatinine, oxygenation indices, C-reactive protein, and procalcitonin; (3) clinical severity assessments, including the Glasgow Coma Scale (GCS) score, Sequential Organ Failure Assessment (SOFA) score, Acute Physiology and Chronic Health Evaluation II (APACHE II) score, and Simplified Acute Physiology Score II (SAPS II).

In this prospective study, all necessary clinical and laboratory data were systematically collected to allow calculation of severity scores (SOFA, APACHE II, and SAPS II) using the established algorithms available on MDCalc (https://www.mdcalc.com/).

Parameter assessment was conducted on the first and fifth days after the diagnosis of sepsis or septic shock in the ICU, following the methodology and findings of the IRIS-7 trial [20].

For CBC and immunophenotyping venous blood samples were collected in K2 EDTA tubes. CBC was performed using a Sysmex XN-1000 analyser (Sysmex Europe GmbH). From patients’ serum CRP (turbidimetry), ferritin (electrochemiluminescence immunoassay ECLIA) and PCT (chemiluminescent immunoassay, CLIA) were determined using Cobas c 501 analyser (Roche Diagnostics).

PLR was obtained by dividing the platelet count to the lymphocyte count as follows:

PLR = platelet count (× 10^3^/μL) / lymphocyte count (× 10^3^/μL)

NLMR was obtained by dividing the neutrophil count by the product of the lymphocyte count and monocyte count and was calculated as follows:

NLMR = neutrophil count (× 10^3^/μL) / [lymphocyte count (× 10^3^/μL) × monocyte count (× 10^3^/μL)]

### Statistical Analysis

All collected data were organised and managed using Microsoft Excel (Microsoft^®^ Excel^®^ for Microsoft 365 MSO Version 2406 Build 16.0.17726.20078, Microsoft Corporation, Redmond, WA, USA). Statistical evaluation, including both descriptive and inferential analyses, was subsequently performed with GraphPad Prism version 8.4.3 (686), released on 10 June 2020 (GraphPad Software, San Diego, CA, USA).

Descriptive statistics included the computation of means or medians with corresponding confidence intervals. The Kolmogorov-Smirnov test was performed to verify the normality of the data distribution. The mean was determined for data exhibiting a normal distribution, while for non-Gaussian distributions, the median and the interquartile range were calculated. Comparisons of paired variables were performed using either the Student’s t-test or the Wilcoxon signed-rank test, selected according to data normality. A *p*-value ≤ 0.05 was regarded as statistically significant.

For receiver operating characteristic (ROC) analysis we used GraphPad Prism version 8.4.3 (686).

## Results

### Population Analysis

The analysed population consisted of 101 individuals, of whom 38 (37.62%) were females and 63 (62.37%) were males. The mean age of the cohort was 68 years, with ages spanning from 33 to 90 years. Most participants (34) were in the 71–80-year age group (n = 34), followed by patients aged 61–70-year (n = 28); whereas only one patient was between 30–40 years old. The mean body mass index was 28.9 ± 5.6, with recorded values ranging from 15.6 to 49.4. Overall, 75 patients (74.25%) did not survive, while 26 patients (25.74%) were discharged alive. The length of ICU stay had a median duration of 14 days, varying from 1 to 95 days.

Among the enrolled patients, 62 (61.38%) were diagnosed with sepsis, while 39 (38.61%) met criteria for septic shock. The distribution of comorbid conditions and infection sources revealed a predominance of cardiovascular disease (n = 83, 83.91%), followed by renal impairment (n = 70, 69.30%) and chronic respiratory disorders (n = 63, 62.37%). Additional conditions, grouped under the category “Other,” included secondary anaemia, thrombocytopenia, chronic tobacco or alcohol use, eschar formation, acid–base imbalances, multiple organ dysfunction syndrome, hypovolemia, malnutrition, and electrolyte disturbances, as detailed in Supplementary materials Table S1.

**Fig. 1.**
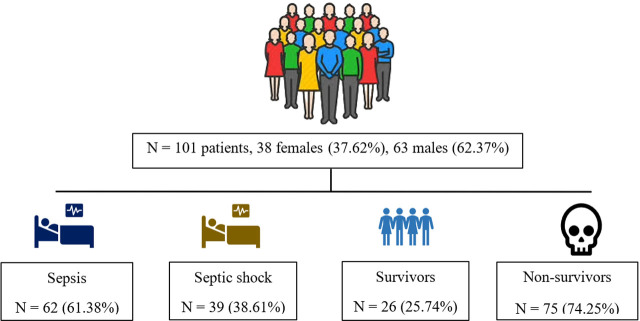
Demographics of the main studied groups

Figure 1 provides the visual representation of the main study group, together with their assigned subgroups. We divided the patients into four subcategories: sepsis and septic shock, survivors and non-survivors.

### Comparison of Variables for the Sepsis and Septic Shock Groups

Patients were categorized into sepsis and septic shock groups based on the Sepsis-3 consensus definitions [2]. The evaluated parameters were assessed on day 1 and day 5 following fulfilment of the inclusion criteria. Detailed descriptive analyses of demographic and clinical characteristics for each group are provided in Supplementary materials Table S2 for patients with sepsis and Table S3 for those with septic shock.

We observed a statistically significant variation for the SOFA score between days 1 and 5 (*p* = 0.0319) for sepsis and for septic shock patients (*p* = 0.0314). No other significant differences were observed (Table 1).

**Table 1.** Comparison of the studied parameters between sepsis and septic shock patients on day 1 and day 5 (median value and interquartile range (IQR)).

**Parameter**	**Sepsis**		**p^a^ value**	**Septic shock**		**p^a^ value**
	
	**Day 1**	**Day 5**		**Day 1**	**Day 5**	
SOFA, points	9 (6.25)	8 (9.25)	**0.0319**	11 (6)	8.5 (10.75)	**0.0314**
APACHE II, points	22 (12.5)	20 (12.25)	0.3137	27 (15)	19.5 (23)	0.9743
SAPS II, points	60.5 (29)	49 (30.5)	0.0875	61 (39)	44.5 (43)	0.8909
NLMR	18.04 (31.94)	13.16 (29.55)	0.8141	33.84 (47.25)	14.01 (22.66)	0.7990
PLR	249.9 (269)	204.2 (222.8)	0.3027	297.7 (309.1)	224.1 (275.3)	0.4319
Carboxyhemoglobin, %	1.3 (0.95)	0.9 (0.9)	0.0944	1.1 (1.2)	1 (1.1)	0.4880
Ferritin, μg/L	393 (481.8)	386 (759)	0.5044	1180 (1250)	521 (859)	0.3926

Legend:

a Wilcoxon test. Bold type indicates significance.

APACHE II – Acute Physiology And Chronic Health Evaluation II, NLMR – Neutrophil-to-Lymphocyte-to-Monocyte Ratio, PLR – Platelet-to-Lymphocyte Ratio, SAPS II – Simplified Acute Physiology Score II, SOFA – Sequential Organ Failure Assessment.

For the sepsis and septic shock groups, correlations between the severity scores pointed out statistically significant positive correlations between SOFA score and APACHE II score, SOFA score and SAPS II score, and between APACHE II score and SAPS II score on both day 1 and day 5 (Table 2).

**Table 2.** Correlations of severity scores for sepsis and septic shock groups on day 1 and day 5

**Parameters**			**APACHE II (points)**	**SAPS II (points)**
SOFA (points)	Sepsis	Day 1	r = 0.6879	r = 0.6865
			(95% CI: 0.529 to 0.8002)	(95% CI: 0.5271 to 0.7992)
			***p*^b^ <0.0001**	***p*^b^ <0.0001**

		Day 5	r = 0.7559	r = 0.7697
			(95% CI: 0.5752 to 0.8663)	(95% CI: 0.5968 to 0.8742)
			***p*^b^ <0.0001**	***p*^b^ <0.0001**

	Septic shock	Day 1	r = 0.7966	r = 0.6953
			(95% CI: 0.6427 to 0.8888)	(95% CI: 0.4866 to 0.829)
			***p*^b^ <0.0001**	***p*^b^ <0.0001**

		Day 5	r = 0.8393	r = 0.8475
			(95% CI: 0.6227 to 0.9364)	(95% CI: 0.6398 to 0.9398)
			***p*^a^ <0.0001**	***p*^a^ <0.0001**

APACHE II (points)	Sepsis	Day 1		r = 0.8227
				(95% CI: 0.7211 to 0.8896)
				***p*^b^ <0.0001**

		Day 5		r = 0.8797
				(95% CI: 0.7791 to 0.9361)
				***p*^b^ <0.0001**

	Septic shock	Day 1		r = 0.8836
				(95% CI: 0.7877 to 0.9377)
				***p*^b^ <0.0001**

		Day 5		r = 0.9575
				(0.8937 to 0.9834)
				***p*^b^ <0.0001**

Legend:

a Spearman test,

b Pearson test.

Bold type indicates significance. APACHE II – Acute Physiology And Chronic Health Evaluation II, SAPS II – Simplified Acute Physiology Score II, SOFA – Sequential Organ Failure Assessment, 95% CI – 95% confidence interval.

**Fig. 2.**
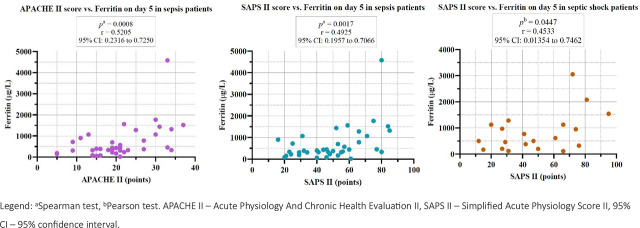
Correlations between APACHE II score and SAPS II score with Ferritin on day 5 for sepsis and septic shock groups.

The severity scores, APACHE II and SAPS II, also correlated positively on day 5 with ferritin for the sepsis and septic shock groups (Figure 2).

Regarding the cellular ratios, in the sepsis group we observed statistically significant correlations on day 1 and day 5 between NLMR and PLR. Also, PLR correlated significantly with ferritin on day 5, and NLMR correlated with ferritin on both studied days. As for the septic shock group, regarding the cellular ratios, we have only observed correlations between PLR and NLMR on day 1, and between NLMR and COHb on day 1 (Table 3).

**Table 3.** Correlations of cellular ratios for sepsis and septic shock patients on day 1 and day 5

**Parameters**			**NLMR**	**COHb (%)**	**Ferritin (μg/L)**
PLR	Sepsis	Day 1	r = 0.3823	r =−0.2078	r =−0.2343
			(95% CI: 0.1391 to 0.582)	(95% CI:−0.4411 to 0.0517)	(95% CI:−0.4632 to 0.024)
			***p*^a^ = 0.0022**	*p*^a^ = 0.1051	*p*^a^ = 0.0669

		Day 5	r = 0.3949	r =−0.1745	r =−0.3216
			(95% CI: 0.09414 to 0.6296)	(95% CI:−0.472 to 0.1587)	(95% CI:−0.5848 to 0.00291)
			***p*^a^ = 0.0097**	*p*^a^ = 0.2881	***p*^a^ = 0.0459**

	Septic shock	Day 1	r = 0.4856	r =−0.2406	r = 0.0534
			(95% CI: 0.1916 to 0.6997)	(95% CI:−0.5169 to 0.08111)	(95% CI:−0.2755 to 0.3712)
			***p*^a^ = 0.0017**	*p*^b^ = 0.1402	*p*^a^ = 0.7466

		Day 5	r = 0.1558	r =−0.1444	r =−0.3063
			(95% CI:−0.3082 to 0.5599)	(95% CI:−0.5517 to 0.3184)	(95% CI:−0.6594 to 0.1576)
			*p*^a^ = 0.4999	*p*^b^ = 0.5435	*p*^b^ = 0.189

NLMR	Sepsis	Day 1		r =−0.1283	r = 0.2989
				(95% CI:−0.3729 to 0.1329)	(95% CI: 0.04561 to 0.5161)
				*p*^a^ = 0.3203	***p*^a^ = 0.0183**

		Day 5		r =−0.0445	r = 0.3542
				(95% CI:−0.3635 to 0.2837)	(95% CI: 0.03387 to 0.6085)
				*p*^a^ = 0.7877	***p*^a^ = 0.027**

	Septic shock	Day 1		r =−0.3976	r = 0.1028
				(95% CI:−0.6394 to −0.0842)	(95% CI:−0.229 to 0.4132)
				***p*^a^ = 0.0122**	*p*^a^ = 0.5333

		Day 5		r = 0.3455	r = 0.05521
				(95% CI:−0.1148 to 0.6836)	(95% CI:−0.3972 to 0.4861)
				*p*^a^ = 0.125	*p*^a^ = 0.8121

Legend:

a Spearman test,

b Pearson test.

Bold type indicates significance. COHb – Carboxyhaemoglobin, NLMR – Neutrophil-to-Lymphocyte-to-Monocyte Ratio, PLR – Platelet-to-Lymphocyte Ratio, 95% CI – 95% confidence interval.

**Fig. 4.**
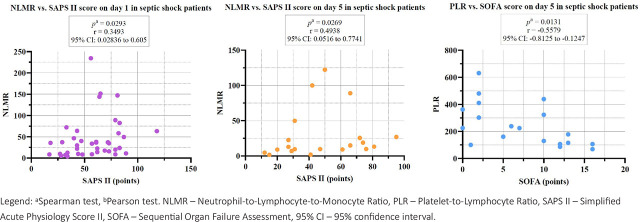
Correlations between cellular ratios and severity scores on day 1 and 5 in the septic shock group.

In the septic shock group, we have observed a statistically significant negative correlation between PLR and the SOFA score on day 5, and a positive correlation between NLMR and SAPS II score on day 1 and 5 (Figure 4).

In the sepsis group, on day 1, we found a statistically significant positive correlation between COHb and ferritin (r = 0.2937, (95% CI: 0.03993 to 0.5119), ***p*^a^ = 0.0205,** ^a^ Spearman test).

### Comparison of Variables for the Survivor and Non-Survivor Groups

The studied population was stratified into survivor and non-survivor groups, and analysed parameters were evaluated on day 1 and day 5 following inclusion. Comprehensive descriptive statistics for each subgroup are presented in Supplementaru Table S4 for survivors and Table S5 for non-survivors. Changes in the assessed parameters between the two time points were studied.

We observed a statistically significant variations only in the survivor group, for the SOFA score (*p* = 0.0028), and SAPS II (*p* = 0.0335). No other variation was significant (Table 4).

**Table 4.** Comparison of the studied parameters between survivors and non-survivor patients on day 1 and day 5 (median value and interquartile range (IQR)).

**Parameter**	**Survivors**		**p^a^ value**	**Non-survivors**		**p^a^ value**
	
	**Day 1**	**Day 5**		**Day 1**	**Day 5**	
SOFA, points	6 (6)	2 (5.75)	**0.0028**	11 (5)	10 (8)	0.0795
APACHE II, points	14 (11.5)	10.5 (10.75)	0.0942	27 (12)	22.5 (16)	0.9814
SAPS II, points	36 (28.5)	28 (16)	**0.0335**	65 (26)	54 (29)	0.6534
NLMR	11.22 (23.54)	9.722 (19.41)	0.8906	23.74 (48.65)	14.88 (28.28)	0.5236
PLR	257 (286.7)	224.8 (308.5)	0.8906	283.2 (304.9)	185.7 (207.7)	0.1091
Carboxyhemoglobin, %	1.35 (1.42)	1 (0.95)	0.1134	1.1 (1.1)	0.9 (0.875)	0.9928
Ferritin, μg/L	393.5 (714.3)	341.5 (548.7)	0.5958	666 (1296)	458.5 (867)	0.9113

Legend:

a Wilcoxon test.

Bold type indicates significance. APACHE II – Acute Physiology And Chronic Health Evaluation II, NLMR – Neutrophil-to-Lymphocyte-to-Monocyte Ratio, PLR – Platelet-to-Lymphocyte Ratio, SAPS II – Simplified Acute Physiology Score II, SOFA – Sequential Organ Failure Assessment.

For the survivor group, correlations between the severity scores pointed out statistically significant positive correlations between SOFA score and APACHE II score, SOFA score and SAPS II score, and between APACHE II score and SAPS II score on both day 1 and day 5 (Table 5).

**Table 5.** Correlations of severity scores for survivors and non-survivors on day 1 and day 5

**Parameters**			**APACHE II (points)**	**SAPS II (points)**
SOFA (points)	Survivors	Day 1	r = 0.6478	r = 0.6398
			(95% CI: 0.3371 to 0.8313)	(95% CI: 0.3356 to 0.8231)
			***p*^a^ = 0.0003**	***p*^b^ = 0.0004**

		Day 5	r = 0.8628	r = 0.7807
			(95% CI: 0.6320 to 0.9530)	(95% CI: 0.4523 to 0.9227)
			***p*^a^ <0.0001**	***p*^a^ = 0.0005**

	Non-survivors	Day 1	r = 0.6273	r = 0.5823
			(95% CI: 0.4655 to 0.7485)	(95% CI: 0.4081 to 0.7156)
			***p*^b^ <0.0001**	***p*^b^ <0.0001**

		Day 5	r = 0.7256	r = 0.6918
			(95% CI: 0.5409 to 0.8435)	(95% CI: 0.4911 to 0.8227)
			***p*^b^ <0.0001**	***p*^b^ <0.0001**

APACHE II (points)	Survivors	Day 1		r = 0.7618
				(95% CI: 0.5226 to 0.8899)
				***p*^a^ <0.0001**

		Day 5		r = 0.6086
				(95% CI: 0.1461 to 0.8528)
				***p*^a^ = 0.014**

	Non-survivors	Day 1		r = 0.818
				(95% CI: 0.7258 to 0.8814)
				***p*^b^ <0.0001**

		Day 5		r = 0.9224
				(95% CI: 0.8569 to 0.9586)
				***p*^a^ <0.0001**

Legend:

a Spearman test,

b Pearson test.

Bold type indicates significance. APACHE II – Acute Physiology And Chronic Health Evaluation II, SAPS II – Simplified Acute Physiology Score II, SOFA – Sequential Organ Failure Assessment, 95% CI – 95% confidence interval.

**Fig. 5.**
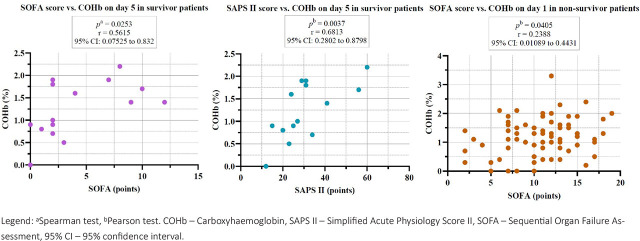
Correlations between severity scores and COHb on day 1 and day 5 in the survivor and non-survivor groups.

We also observed statistically significant positive correlations between SOFA score and SAPS II score with COHb on day 5 in the survivor group, and a positive correlation between SOFA score and COHb on day 1 in the non-survivor group (Figure 5).

**Fig. 6.**
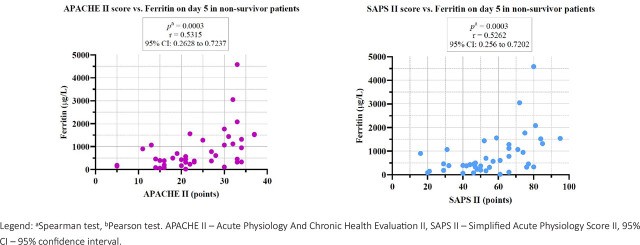
Correlations between severity scores and ferritin on day 5 in the non-survivor group.

In the non-survivor group, we found statistically significant positive correlations on day 5 between APACHE II with ferritin and SAPS II with ferritin (Figure 6).

Regarding the cellular ratios, we observed statistically significant positive correlations between PLR and NLMR on both day 1 and day 5 for the survivors group. Also, NLMR correlated statistically with ferritin on both study days. For no-survivors, we observed a positive correlation between PLR and LNMR, and PLR and COHb on day 1. On day 5, we observed correlations between PLR and ferritin on day 5, NLMR and ferritin on day 1 (Table 6).

**Table 6.** Correlations of cellular ratios for survivor and non-survivor patients on day 1 and day 5

**Parameters**			**NLMR**	**COHb (%)**	**Ferritin (μg/L)**
PLR	Survivors	Day 1	r = 0.4099	r = −0.2135	r = −0.1768
			(95% CI: 0.01474 to 0.6943)	(95% CI: −0.5633 to 0.2011)	(95% CI: −0.5366 to 0.2375)
			***p*^a^ = 0.0375**	*p*^a^ = 0.2949	*p*^a^ = 0.3877

		Day 5	r = 0.6351	r = −0.0295	r = 0.1517
			(95% CI: 0.2406 to 0.8495)	(95% CI: −0.4897 to 0.4434)	(95% CI: −0.3524 to 0.5875)
			***p*^a^ = 0.0035**	*p*^b^ = 0.9072	*p*^a^ = 0.5479

	Non-survivors	Day 1	r = 0.4255	r = −0.3133	r = −0.07673
			(95% CI: 0.2133 to 0.5994)	(95% CI: −0.5095 to −0.08616)	(95% CI: −0.3047 to 0.1596)
			***p*^a^ = 0.0001**	***p*^a^ = 0.0062**	*p*^a^ = 0.5129

		Day 5	r = 0.1619	r = −0.1762	r = −0.4354
			(95% CI: −0.1507 to 0.445)	(95% CI: −0.4664 to 0.1481)	(95% CI: −0.6606 to −0.1383)
			*p*^a^ = 0.2938	*p*^a^ = 0.2704	***p*^a^ = 0.0044**

NLMR	Survivors	Day 1		r = −0.1283	r = 0.1275
				(95% CI: −0.3729 to 0.1329)	(95% CI: −0.2845 to 0.4998)
				*p*^a^ = 0.3203	*p*^a^ = 0.5347

		Day 5		r = −0.0445	r = 0.3168
				(95% CI: −0.3635 to 0.2837)	(95% CI: −0.1906 to 0.6906)
				*p*^a^ = 0.7877	*p*^a^ = 0.2002

	Non-survivors	Day 1		r = −0.1951	r = 0.2358
				(95% CI: −0.4099 to 0.04015)	(95% CI: 0.002498 to 0.4447)
				*p*^a^ = 0.0935	***p*^a^ = 0.0417**

		Day 5		r = 0.1046	r = 0.1733
				(95% CI: −0.2148 to 0.4037)	(95% CI: −0.147 to 0.4607)
				*p*^a^ = 0.5099	*p*^a^ = 0.2723

Legend:

a Spearman test,

b Pearson test.

Bold type indicates significance. COHb – Carboxyhaemoglobin, NLMR – Neutrophil-to-Lymphocyte-to-Monocyte Ratio, PLR – Platelet-to-Lymphocyte Ratio, 95% CI – 95% confidence interval.

**Fig. 7.**
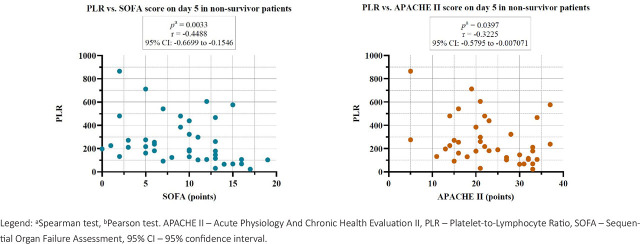
Correlations between PLR and severity scores on day 5 in the non-survivor group.

Furthermore, in the non-survivor group we observed statistically significant negative correlations between PLR and SOFA score, and PLR and APACHE II on day 5 of study inclusion (Figure 7).

Table 7 offers information regarding the lowest and highest value of ferritin for each subgroup of patients, and classification of patients according to their number.

**Table 7.** Patient classification according to ferritin value, with lowest and highest value for each subgroup

	**Intervals (μg/L)**	**Sepsis (N = 62; 41)**	**Septic shock (N = 39; 21)**	**Survivors (N = 26; 20)**	**Non-survivors (N = 75; 42)**	**Reference interval**
Day 1	0–20	2 (9.53 μg/L)	0	0	2 (9.53 μg/L)	20–290 μg/L
	21–100	5	2 (54.9 μg/L)	2 (44.3 μg/L)	5	
	101–200	6	4	1	9	
	201–290	6	1	3	4	
	> 291	45 (6220 μg/L)	32 (7300 μg/L)	20 (2700 μg/L)	55 (7300 μg/L)	

Day 5	0–20	1 (20.6 μg/L)	0	0	1 (20.6 μg/L)	
	21–100	3	0	0	3	
	101–200	5	5 (110 μg/L)	5 (111 μg/L)	5	
	201–290	2	0	1	1	
	> 291	30 (4580 μg/L)	16 (3050 μg/L)	14 (2720 μg/L)	32 (4580 μg/L)	

### Predictive Performance of Clinical Scores and Biomarkers for Mortality

We evaluated the predictive performance of the studied clinical scores (SOFA, APACHE II, SAPS II) and inflammatory biomarkers (NLMR, PLR, carboxyhaemoglobin, and ferritin) in relation to in-hospital mortality. To determine the area under the curve (AUC) for each parameter and to identify optimal cutoff values based on Youden’s index, we performed receiver operating characteristic (ROC) curve analysis. For each variable, sensitivity, specificity, confidence intervals, and statistical significance were calculated. These analyses were conducted separately for values obtained on day 1 (study inclusion) and day 5, allowing assessment of changes in prognostic accuracy over time.

**Fig. 8.**
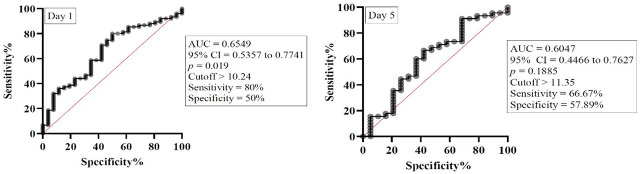
ROC curve for NLMR on day 1 and day 5. Legend: AUC – Area under curve, 95% CI – 95% confidence interval.

The NLMR measured on day 1 showed modest discriminatory ability for mortality, with an AUC of 0.6549 (95% CI: 0.5357 to 0.7741, *p* = 0.019) and a cutoff value >10.24 (sensitivity: 80% and specificity: 50%). However, by day 5, predictive performance decreased (AUC: 0.6047, 95% CI: 0.4466 to 0.7627, *p* = 0.1885), with a cutoff value > 11.35 (sensitivity: 66.67% and specificity: 57.89%) (Figure 8).

**Fig. 9.**
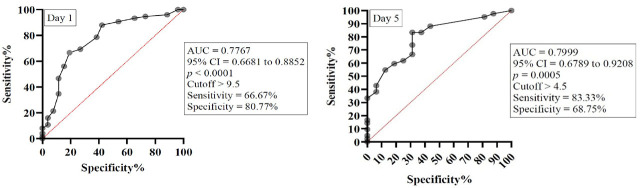
ROC curve for SOFA score on day 1 and day 5. Legend: AUC – Area under curve, 95% CI – 95% confidence interval.

The SOFA score showed a good ability to predict mortality both at inclusion and on day 5. On day 1, the AUC was 0.7767 (95% CI 0.6681 to 0.8852, p < 0.0001), with an optimal cutoff value > 9.5, corresponding to a sensitivity of 66.67% and specificity of 80.77%. On day 5, the AUC increased to 0.7999 (95% CI 0.6789 to 0.9208, p = 0.0005), with a cutoff > 4.5 (sensitivity 83.33%, specificity 68.75%), suggesting a consistent and significant predictive performance over time (Figure 9).

**Fig. 10.**
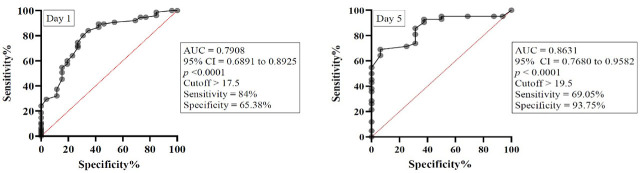
ROC curve for APACHE II score on day 1 and day 5. Legend: AUC – Area under curve, 95% CI – 95% confidence interval.

The APACHE II score also demonstrated significant discriminative power for mortality. On day 1, the AUC was 0.7908 (95% CI 0.6891 to 0.8925, p < 0.0001), with an optimal cutoff > 17.5 (sensitivity 84%, specificity 65.38%). On day 5, predictive accuracy improved further, with an AUC of 0.8631 (95% CI 0.7680 to 0.9582, p < 0.0001) and an optimal cutoff > 19.5 (sensitivity 69.05%, specificity 93.75%), indicating strong prognostic capability (Figure 10).

**Fig. 11.**
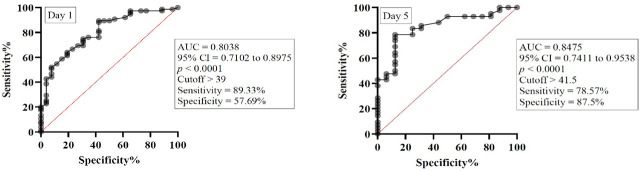
ROC curve for SAPS II score on day 1 and day 5. AUC – Area under curve, 95% CI – 95% confidence interval.

Similarly, the SAPS II score exhibited good predictive ability. On day 1, the AUC was 0.8038 (95% CI 0.7102 to 0.8975, p < 0.0001), with an optimal cutoff > 39 (sensitivity 89.33%, specificity 57.69%). On day 5, the AUC was 0.8475 (95% CI 0.7411 to 0.9538, p < 0.0001), and the best cutoff was > 41.5 (sensitivity 78.57%, specificity 87.5%), confirming high accuracy in predicting mortality (Figure 11).

PLR, carboxyhaemoglobin, and ferritin did not show significant discriminative ability for mortality (AUC < 0.65, p > 0.05) and thus were not considered strong individual predictors in this cohort.

## Discussions

In earlier centuries, the spread of infectious diseases worldwide was often facilitated by colonization, slavery, and warfare. Before vaccines became available, diseases such as tuberculosis, polio, smallpox, and diphtheria were a leading cause of death and suffering. Significant improvements over the last twenty years in public health outcomes due to advancements in medical care, wider access to health services, and better sanitation practices have all contributed to reducing mortality and morbidity associated with infectious diseases [21].

A 2017 global study regarding sepsis morbidity and mortality, estimated 49 million sepsis cases and 11 million related deaths, accounting for 20% of annual mortality. Lower-middle-income countries had the highest burden. The average hospital cost per case exceeded USD 32.000, mainly in high-income countries [3, 22]. In response to the growing global impact, the WHO released its first report on sepsis in 2020, urging professionals to strengthen efforts to improve sepsis surveillance and reporting [4]. Sepsis remains closely tied to systemic weaknesses such as limited access to quality care, under-resourced health systems, poor infection control, delayed recognition, and inadequate clinical response [4, 23]. According to the 2020 WHO report, in the European region alone, mortality reached 42.7%, with an incidence of 139 cases per 100.000 adults per year [4, 24].

The current definition of sepsis, as a life-threatening organ dysfunction syndrome caused by a dysregulated host response to infection, established by the 2016 Sepsis-3 Consensus, underscores the existence of inflammatory and immunosuppressive states that may occur sequentially or concurrently [2]. The primary goal of the immune system during the early stage of the inflammatory response is to eliminate pathogens and restore homeostasis. Failure to achieve clearance of pathogens results in an imbalance of the immune system, manifesting as secondary infections, long-term immunosuppression, and increased morbidity and mortality [25].

Despite significant advancements in the pathophysiology and immunological mechanisms that occur during the septic episode, there is still much improvement to be made. In recent years, growing attention has been directed toward strategies designed to counteract the immunosuppressive phase of sepsis. Timely and precise evaluation of a patient’s immune status is essential for the early detection of immune dysregulation and for guiding the appropriate initiation of immunomodulatory interventions [25,26,27,28,29].

Severity scores continue to serve as essential tools in intensive care units for evaluating disease severity, monitoring therapeutic response, and estimating the risk of mortality in patients with sepsis or septic shock. Recent studies still debate the predictive accuracy of these scoring models for morbidity and mortality, as they primarily capture the clinical status at a single point in time during the ICU stay. Thus, repeated assessment of the patient’s clinical status using severity scores such as SOFA, APACHE II and SAPS II, either independently or in combination, is recommended to enhance prognostic reliability [30].

In our study, when comparing the severity scores and their variation between the studied days, we observed a statistically significant variation for the SOFA score between day 1 and day 5 for the sepsis, septic shock, and survivor groups. In the survivor group, we also found a statistically significant variation for the SAPS II score between the two studied days. Notably, when dividing the patients into sepsis and septic shock, and survivors and non-survivors, we observed statistically significant positive correlations between the severity scores, SOFA, APACHE II, and SAPS II, on both day 1 and day 5 for all the aforementioned categories. A study fom 2024 published by Liengswangwong W. et al. found that the SOFA score was the optimal predictor of septic shock, when compared to other severity screening scores, such as APACHE II and SAPS II [31]. However, these screening tools are limited, due to the large variability in patient population, offering only indirect insights into the extent of multiple organ dysfunction present at a given stage in the progression of sepsis [32, 33].

Iron is an essential element of haemoproteins, including haemoglobin, myoglobin, and cytochromes involved in the electron transport chain [34]. Maintaining iron homeostasis is of paramount importance, as iron deficiency can impair oxidative phosphorylation, compromise oxygen transport, and contribute to metabolic failure. Conversely, iron overload promotes the formation of reactive oxygen species, leading to oxidative damage and the degradation of essential cellular proteins [16, 34]. Ferritin functions as an acute-phase reactant; serum values reflect both acute and chronic inflammatory conditions associated with various conditions, including infectious, autoimmune, hematologic, and malignant diseases. Elevated serum ferritin levels are frequently observed in patients with sepsis and septic shock, reflecting the presence of ongoing systemic inflammation.

In our study, we observed statistically significant positive correlations between severity scores, APACHE II and SAPS II, with ferritin on day 5 of study inclusion for septic patients, septic shock patients, and non-survivor patients, suggesting that higher ferritin levels are associated with increased illness severity and mortality risk. Furthermore, as shown in Table 7, the majority of patients, regardless of the study group, presented ferritin values higher than 290 μg/L, indicating an intensified inflammatory and metabolic response associated with increased clinical severity. Our findings align with recent literature, a study from 2022 by Sang L. and collab. demonstrated that ferritin serves not only as an acute-phase reactant reflecting systemic inflammation, but it could also serve as predictive marker of clinical outcomes [35].

Elevated ferritin levels, regardless of the primary aetiology, have been consistently associated with an increased mortality risk [34]. The correlation with severity scores suggests it parallels physiological derangements captured by APACHE II and SAPS II.

Patients afflicted by sepsis suffer persistent infections, organ dysfunction, prolonged immunosuppression, and ultimately, death. Sepsis onset triggers the activation of cells belonging to the innate immune system, including neutrophils, monocytes, macrophages, and natural killer cells [1, 36,37,38]. The exact timing at which adaptive immunity becomes actively involved in the host response to sepsis remains unclear. However, findings from our previous investigations have demonstrated a marked reduction in T lymphocyte subsets, specifically helper T cells (CD4^+^) and cytotoxic T cells (CD8^+^), as early as day 1 following the clinical diagnosis of sepsis or septic shock. This supports our hypothesis that lymphocyte apoptosis plays a central role in the immunopathogenesis of both conditions. Additionally, in patients with septic shock, we observed a subsequent increase in CD8^+^ T cell counts from day 1 to day 5, indicative of an intensified pro-inflammatory response [7, 39].

Recent literature focused on assessing the innate immunity through a series of ratios between the cellular components of it. Thus, the NLR has been used as a prognostic biomarker in sepsis and serves as a marker of the interplay between innate and adaptive immunity, emerging as a reliable indicator of systemic inflammation and physiological stress [9, 10]. Inflammatory and coagulation cascades in sepsis promote platelet activation, while lymphopenia, a hallmark of sepsis-associated immunosuppression, impairs microbial clearance and increases susceptibility to secondary infections. These two parameters are integrated in the PLR, reflecting the interplay between thrombotic and inflammatory responses [11]. Monocytes play a critical role in maintaining cellular homeostasis during infection and inflammation through the removal of pathogens, foreign substances, and apoptotic or necrotic cells [12]. In this context, the LMR has been investigated as a potential predictive biomarker for sepsis [13]. Previous studies evaluating the prognostic value of NLR, PLR, and LMR in sepsis have showed that these cellular ratios may predict the presence of systemic inflammatory responses and sepsis. More recently, Lin et al. demonstrated that NLMR offers a rapid and direct indicator for predicting early mortality risk in adult patients with septic shock [1, 13].

In the present study, we observed statistically significant positive correlations between NLMR and PLR in all the studied groups. These correlations were present on both day 1 and day 5 in patients from the sepsis group and for the survivors group, and on day 1 in patients with septic shock and in non-survivors. This finding reflects the interconnected dynamics of cellular immune responses during sepsis and septic shock. The observed positive correlation between PLR and NLMR suggests that these ratios may rise in parallel during systemic inflammation, reflecting a combined effect of increased innate immune activity and suppressed adaptive immunity. In patients with sepsis and in survivors, the persistence of this correlation at both time points may indicate an immune response that, while activated, remains partially regulated.

A recent 2022 meta-analysis discussing the prognostic value of PLR in sepsis found that inflammation is linked to PLR values; significantly higher levels of PLR were found in non-survivors, supporting our findings, stating that the presence of the correlation only on day 1 in patients with septic shock and in non-survivors may reflect an initial overwhelming immune activation that ultimately progresses toward immunoparalysis or organ failure [11, 40]. Furthermore, a 2024 study by Lin and collab. assessing the application of NLMR in predicting mortality of patients with septic shock found that an increased neutrophil count was associated with a higher risk of early death in adult patients with septic shock. The study also found that NLMR was more accurate in predicting mortality than SOFA and APACHE II scores [1]. In a previous study that we published, we observed lymphopenia in all the studied patient categories from day 1 of study inclusion, compared to standard laboratory values, which was explained by the migration of active lymphocytes to the area of inflammation, as well as lymphocyte apoptosis [40, 41].

In our study, a negative correlation was observed between the PLR and serum ferritin levels on day 5 in patients with sepsis. This inverse relationship may suggest a decoupling between platelet-driven inflammation and iron-mediated acute-phase responses as the disease progresses. A decreasing PLR alongside persistently elevated ferritin levels could indicate immune exhaustion or a transition to an immunosuppressive state later in the disease course [17]. Conversely, NLMR demonstrated a positive correlation with ferritin levels on both day 1 and day 5 in sepsis patients, highlighting the association between persistent innate immune activation and increased ferritin concentrations. Similarly, in the non-survivor group, a negative correlation was observed between PLR and ferritin on day 5, while a positive correlation between NLMR and ferritin was noted on day 1. These findings reinforce the role of ferritin as a biomarker of disease severity and underline the utility of combined inflammatory ratios in evaluating the host immune response in critical illness. Thus, hyperferritinemia reflects heightened activation of the innate immune system, as ferritin is both an acute-phase reactant and a mediator of inflammation, released predominantly by activated macrophages in response to pro-inflammatory cytokines [17].

Although not specific to sepsis, COHb is an indirect marker of endogenous CO production by the liver, mediated by the HO-1 pathway. Its production increases in response to oxidative stress and systemic inflammation. Sepsis, characterised by impaired heme metabolism and hepatic dysfunction, contributes to elevated COHb levels, driven by factors such as oxidative injury, tissue hypoxia, proinflammatory cytokines, and endotoxins [42, 43].

In the septic shock group, we observed a statistically significant negative correlation between NLMR and COHb levels on the first day of study inclusion. Similarly, within the non-survivor group, a negative correlation was identified between PLR and COHb on day 1. These inverse relationships may reflect the complex interplay between immune cell activation, oxidative stress, and cellular hypoxia in the context of advanced septic states, where elevated inflammatory indices overlap with impaired tissue oxygenation and CO metabolism. As COHb is routinely measured in arterial blood gas analysis in the ICU, significant shifts in its levels may provide early clinical insight into evolving disease dynamics [15].

Related to COHb, we observed correlations with severity scores. Statistically significant positive correlations were observed between COHb levels and both the SOFA and SAPS II scores on day 5 in the survivor group, suggesting an association between COHb and the degree of organ dysfunction. Additionally, a positive correlation was identified between the SOFA score and COHb levels on day 1 in the non-survivor group, indicating that elevated COHb may reflect early severity of illness and predict adverse outcomes. A 2023 study by Dani C and collab. found that increasing COHb levels up to two days before the diagnosis were significantly associated with increased risk of sepsis onset [44]. Also, on day 1, in the sepsis group, we observed a statistically significant positive correlation between COHb levels and serum ferritin, suggesting a possible link between oxidative stress and systemic inflammation in the early phase of sepsis.

In our study NLMR demonstrated modest but significant prognostic value at admission, reflecting early immune dysregulation in sepsis, but lost predictive accuracy by day 5. These findings suggest that NLMR is best suited as an early adjunct biomarker rather than a standalone or longitudinal predictor of mortality. On the other hand, SOFA, APACHE II, and SAPS II scores showed consistent predictive performance for mortality at both admission and day 5, reflecting their ability to quantify cumulative organ dysfunction and physiological derangement in sepsis. Our findings are consistent to those of Zhang and collab. who found that NLMR was a useful prognostic tool for early mortality in patients with severe pneumonia [45].

This study presents several limitations. The study was conducted in a single intensive care unit, which may limit the general applicability of the findings to other settings with different patient populations, management protocols, or resource availability. Due to the relatively small sample size, the study may lack the statistical power needed to detect more subtle correlations or to generalize its findings to broader populations. Measurements of biomarkers and scores were limited to specific time points. Continuous monitoring might provide a more accurate reflection of the dynamic changes in sepsis progression. In the future, the authors plan to expand the study group and the timeframe of biomarker monitoring to encompass additional days, which may provide deeper insight into the prognostic trends of biomarkers throughout sepsis and septic shock.

## Conclusions

This study highlights the clinical significance of cellular ratios (NLMR and PLR) and severity scores in the prognostic evaluation of patients with sepsis and septic shock. The correlations found between ferritin and severity scores, particularly in the sepsis and non-survivor groups, support the statement that ferritin reflects systemic inflammation and disease severity.

In addition, the correlations found between cellular ratios and COHb and ferritin in all the studied groups reveal complex interactions between immune dysregulation, oxidative stress, and organ dysfunction. Furthermore, the correlations found between COHb and severity scores, in both survivor and non-survivor groups, reflect systemic stress and inflammation, and can enhance the clinical image of disease severity, especially when measured frequently.

While SOFA, APACHE II, and SAPS II provided robust mortality prediction, inflammatory biomarkers such as NLMR offered additional early insight but limited standalone prognostic value for our lot of patients.

Importantly, the studied parameters are cost-effective, readily available, and feasible to monitor at the bedside, making them valuable adjuncts in routine ICU care. In contrast, ferritin, although informative, is less practical for repeated monitoring due to higher costs and laboratory requirements.

Therefore, combining routinely accessible tools with targeted biomarkers may provide a balanced, resource-sensitive approach to individualized risk stratification in sepsis. The combination of the aforementioned biomarkers is suggesting potential utility, though further multicentric prospective studies are needed.
